# Opportunities, challenges and future perspectives of using bioinformatics and artificial intelligence techniques on tropical disease identification using omics data

**DOI:** 10.3389/fdgth.2024.1471200

**Published:** 2024-11-25

**Authors:** S. M. Vidanagamachchi, K. M. G. T. R. Waidyarathna

**Affiliations:** ^1^ Department of Computer Science, Faculty of Science, University of Ruhuna, Matara, Sri Lanka; ^2^ Department of Information Technology, Sri Lanka Institute of Advanced Technological Education, Galle, Sri Lanka

**Keywords:** genomics, transcriptomics, proteomics, tropical, bioinformatics, AI, vision transformers, advanced AI

## Abstract

Tropical diseases can often be caused by viruses, bacteria, parasites, and fungi. They can be spread over vectors. Analysis of multiple omics data types can be utilized in providing comprehensive insights into biological system functions and disease progression. To this end, bioinformatics tools and diverse AI techniques are pivotal in identifying and understanding tropical diseases through the analysis of omics data. In this article, we provide a thorough review of opportunities, challenges, and future directions of utilizing Bioinformatics tools and AI-assisted models on tropical disease identification using various omics data types. We conducted the review from 2015 to 2024 considering reliable databases of peer-reviewed journals and conference articles. Several keywords were taken for the article searching and around 40 articles were reviewed. According to the review, we observed that utilization of omics data with Bioinformatics tools like BLAST, and Clustal Omega can make significant outcomes in tropical disease identification. Further, the integration of multiple omics data improves biomarker identification, and disease predictions including disease outbreak predictions. Moreover, AI-assisted models can improve the precision, cost-effectiveness, and efficiency of CRISPR-based gene editing, optimizing gRNA design, and supporting advanced genetic correction. Several AI-assisted models including XAI can be used to identify diseases and repurpose therapeutic targets and biomarkers efficiently. Furthermore, recent advancements including Transformer-based models such as BERT and GPT-4, have been mainly applied for sequence analysis and functional genomics. Finally, the most recent GeneViT model, utilizing Vision Transformers, and other AI techniques like Generative Adversarial Networks, Federated Learning, Transfer Learning, Reinforcement Learning, Automated ML and Attention Mechanism have shown significant performance in disease classification using omics data.

## 1 Introduction

Tropical diseases may occur mainly in tropical and subtropical regions throughout the world. These regions include areas close to the equator, which are characterized by hot and humid climates. These diseases can often be caused by viruses, bacteria, parasites, and fungi (pathogens). Further, they are spread through vectors (i.e., mosquitoes, flies, and other insects). The main reason for the prevalence of these diseases is poor sanitation, inadequate and limitations in healthcare facilities, and poverty. For example Malaria, Dengue fever, chikungunya virus, Zika virus and yellow fever can be considered as tropical diseases.

Omics data include various studies of entire set of genes, RNAs, proteins and metabolites of various organisms are known as genomics, transcriptomics, proteomics and metabolomics respectively. **Genomics** data types include DNA sequences, single nucleotide polymorphisms (SNPs), copy number variations (CNVs), transcriptomics include mRNA, non-coding RNAs (e.g., miRNA, lncRNA), RNA-seq data, **proteomics** include protein sequences, post-translational modifications, protein-protein interactions, and **metabolomics** include metabolic profiles, metabolite concentrations. Out of various genomics applications, identifying genetic variations associated with diseases, evolutionary studies, and personalized medicine are a few applications. Understanding gene expression patterns, identifying gene regulatory networks, studying the effects of environmental factors on gene expression are some of the applications on transcriptomics. Some of the applications on proteomics are disease biomarker discovery, protein function and interaction identification, and drug target identification. Further, studying metabolic pathways, disease diagnostics, understanding the biochemical effects of drugs and toxins are a few applications in metabolomics. Integration of multiple omics data types can provide comprehensive understanding and insights into biological system functions, disease progression (i.e., cancers, infectious diseases, neurological disorders, etc.).

Early detection of tropical diseases is critical for effective treatments. Bioinformatics tools and diverse AI techniques are pivotal in identifying and understanding tropical diseases through the analysis of omics data. There exist several tools for bioinformatics analysis of genomics, transcriptomics, proteomics and metabolomics, as well as integrated multi-omics data. These tools can combine to enhance the ability to identify, understand, and predict tropical diseases by providing comprehensive analyses and insights of various omics data types. Wide range of AI techniques has significantly improved the field of tropical disease identification. Among them unsupervised and supervised Machine Learning (ML), Deep Learning (DL), Natural Language Processing (NLP) with GenAI and LLMs, Ensemble ML and Deep Ensemble Learning (DEL), and the most recent advances such as Explainable AI, Responsible AI etc. Various AI models can be trained on omics data to perform disease prediction, progression, and treatment response. These models can also be utilized to identify potential biomarkers for early diagnosis of tropical diseases and targeted therapy (1).

### 1.1 Road map

The rest of the article is organized as follows: Section 2 discusses the research methods and the process of the study. Section 3 then presents the literature review of both Bioinformatics and AI technology utilization in predicting tropical diseases using different omics datasets. It discusses the opportunities and challenges to predict tropical diseases with omics based AI and Bioinformatics technologies. Sections 4, 5 concludes the article, and suggests some future directions respectively.

## 2 Research methods and process

**Figure 1:**
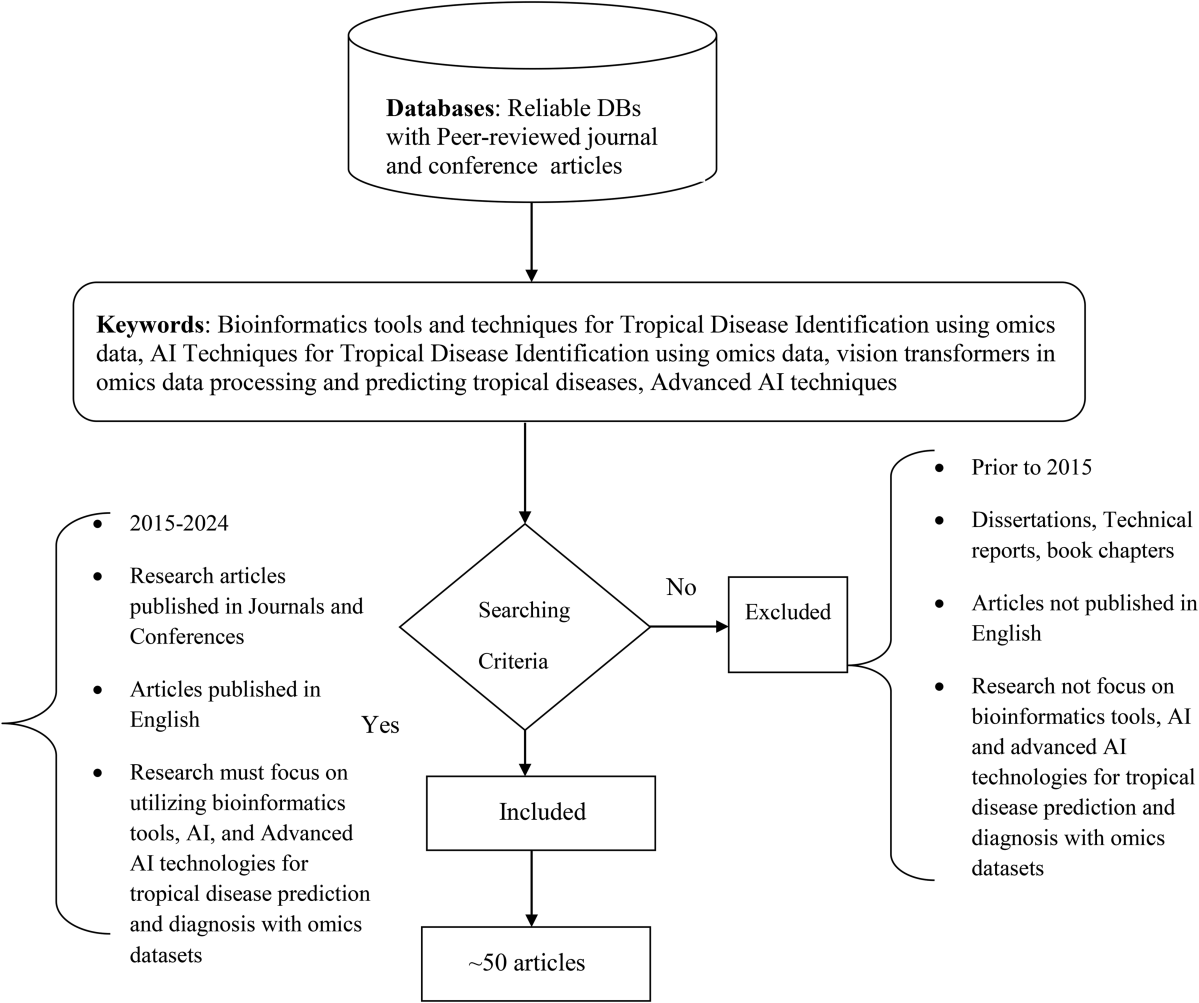
Flow diagram of the research process.

In this section, we provide details of our research methodology. Figure 1 indicates our research process. We have explored research papers using both extensive and explicit search approach. We selected research papers based on keywords, year of publication, their utilization of encompass Bioinformatics tools and AI techniques for tropical disease identification with omics data. We reviewed our selected papers to identify a few important questions and their answers. This article presents a comprehensive analysis, in which we describe Research Questions (RQs), and provide a detailed description of the answers to the questions. Our contribution is novel in identifying research questions, analyzing the opportunities and challenges, describing a detailed review of literature, and outlining future directions for research. Following are the major research questions we addressed throughout this paper.

(1) What are the key findings of state-of-the-art bioinformatics and AI-enabled techniques on tropical disease identification using omics data?
(2) What are the opportunities and challenges in current bioinformatics and AI-enabled systems specially using Advanced AI techniques?
(3) What are the future research directions of bioinformatics and AI-enabled systems to effectively use them in tropical disease identification?

Answers to the above mentioned questions are based on an extensive review of the existing literature, in which we collected and discussed information and knowledge related to Bioinformatics tools and techniques, AI-enabled techniques including the recent advances. In addition, we have also discussed limitations, challenges, and future directions of bioinformatics and AI-enabled techniques with recent advancements including transformer-based implementations (i.e., language models and vision transformers).

We initiate our research process by thoroughly formulating research questions and designing specified search strategies. Subsequently, we rigorously evaluate search results, adhering strictly to predetermined inclusion and exclusion criteria. Once we've selected relevant articles, we delve into their content, synthesizing our findings cohesively. Finally, we present our discoveries and present discussions. Here are the inclusion criteria guiding our research

1. Articles published within the last ten years (2015–2024) to capture recent advancements and developments in the field.
2. Research articles published in Journals and Conferences are considered
3. Research must focus on utilizing bioinformatics tools and AI technologies for tropical disease prediction and diagnosis with omics datasets
4. Only the articles published in English are considered.

Furthermore, we defined exclusion criteria for this systematic literature review to ensure that only associated and high-quality studies were included. Studies that meet the following criteria have been excluded from the review:

1. Articles published before 2015
2. Dissertations, Technical reports, and book chapters were excluded. In addition, articles that were not peer-reviewed, such as blog posts, opinion pieces, or other informal publications, were excluded to maintain the academic rigor of the review.
3. Studies that have not been published in English were excluded. While this can limit the inclusion of some relevant research, it ensures that all included studies are fully understood and accurately assessed by the reviewers.
4. Duplicate studies, or those with overlapping content, were identified and only the most comprehensive version was included. This was to avoid redundancy and ensure a wide range of perspectives and findings.
5. Research not focusing on utilizing bioinformatics tools and AI technologies for tropical disease prediction with omics datasets
6. Studies that lacked sufficient methodological detail citations, making it impossible to judge the reliability and validity of the findings, were excluded. This included studies with vague descriptions of their experimental or analytical methods.

By applying and choosing these exclusion criteria, we aimed to ensure that our systematic literature review includes only the most relevant and high-quality studies. This approach enhances the reliability and validity of the findings of this review, contributing a solid foundation for understanding how Bioinformatics tools and AI techniques provide insights into tropical diseases utilizing various omics data.

## 3 Literature review

Bioinformatics is a field which combines biology, computer science, and statistics to analyze various types of biological data. It plays a critical role in understanding complex biological processes, including disease mechanisms. Predictive modeling, often supported by Machine Learning (ML) techniques, is a key component of bioinformatics. Further, bioinformatics tools and techniques are highly utilized in tropical disease predication and identification with the input of omics datasets. In addition, wide flavors of AI play vital roles in different areas such as medicine, engineering, biology, linguistics, psychology, pharmacology, education, and neuroscience. Firstly, this review focuses on how Bioinformatics techniques bring transformative opportunities in the prediction and diagnosis of tropical diseases with the support of omics data. Then it investigates how AI can be utilized in advanced in the same purpose in more complex problem solving (2). Following section presents our main contributions of the systematic review.

### 3.1 Opportunities and challenges/limitations using bioinformatics and AI techniques for disease predictions

Though there are several improvements and advances in the field of disease prediction, diagnosis, predictive modeling etc. utilizing omics datasets including multi-omics data integration, there exist challenges and limitations of them allowing some room for the improvements.

Table 1 presents opportunities, challenges and future recommendations of past research on utilizing Bioinformatics tools for Omics Data Processing for disease predictions and Table 2. The opportunities, challenges and future recommendations utilizing suitable AI technologies with Omics data (at the end of the paper).

**Table 1:** Opportunities, challenges and future recommendations of past research on utilizing bioinformatics tools for omics data processing for disease predictions (including tropical diseases).

Main findings	Opportunities	Challenges	Future recommendations
- Integration of multiple omics data improves disease prediction, biomarker identification, and treatment approaches with the support of ML and AI tecniques - Bioinformatics resources i.e., Databases like GenBank, EuPathDB, and tools like BLAST, Clustal Omega are widely used in infectious disease research (3).	- Enhanced diagnostic tools, early detection methods, real-time monitoring and surveillance of infectious disease outbreaks, through advanced bioinformatics tools and AI-driven platforms. - Integrating patient-specific omics data to enable personalized medicine approaches, improving vaccine development (by identifying novel antigens and immune responses)	- High complexity and variability of omics data with the Integration and standardization require sophisticated computational methods for comprehensive analysis. - Ethical considerations are required for handling sensitive health data. - Access limitations to high-quality, annotated datasets for training AI models.	- Development of standardized protocols for omics data elicitation (including open-access data sharing with privacy considerations) and analysis to ensure consistency and reproducibility - Make collaborations between bioinformaticians, clinicians, etc.to bridge the gap between data analysis and practical usage - Make investments for computational platforms, and training programs to build capacity in technology.
- Utilization of bioinformatics tools and resources has led to considerable advancements in pathogen characterization, vaccine development and tracking pathogen evolution (4).	- Enhanced disease diagnosis for early detection, outbreak prediction. - Personalized medicine and treatments based on genetic profiles. - Potential of conducting large-scale epidemiological studies and public health improvements.	- Limited infrastructure and technical personnel. - Data privacy and ethical problems - Challenge in the integration of diverse datasets. - High cost of sequencing and data analysis.	- Invest in infrastructure and training on bioinformatics tool usage and develop standardized protocols and data-sharing frameworks - Foster collaborations. - Promote utilizing open-access databases and collaborative environments
Integration of multi-omics data allow scientists to identify novel associations between biomolecules and disease phenotypes, helps to establish detailed biomarkers, and search signaling pathways (5).	Multi-omics enables personalized pharmaceutical approaches, probable drug target identification, and therapeutic strategy optimization.	Data integration, experimental design, and the requirement of robust analysis methods are limitations.	More developments of deep learning approaches for multi-omics integration and applications in precision medicine.
- Bioinformatics tools are a must for analyzing and interpreting omics data. - Integration of multi-omics data with AI provides comprehensive insights into pathogen biology and host responses in infectious disease research (6).	- Development of novel diagnostics, therapeutics and identification of novel biomarkers, and drug targets. - Personalized medicine approaches for infectious diseases. - Improved disease surveillance and outbreak prediction	- Handling large, complex datasets. - Issues in data standardization and interoperability. - Limited access to high-quality, and annotated data. - Ethical considerations (including privacy) related to omics data.	- Enhance computational infrastructure and bioinformatics training. - Develop and use standardized protocols for data management. - Promote sharing data and research collaborations. - Establish proper regulations to ensure data privacy and ethical concerns.
- Integration of bioinformatics tools with genomics and proteomics data enhances the efficiency of antigen discovery processes. - High-throughput techniques can be used for the identification and of potential vaccine candidates (7).	- Improving vaccine design via comprehensive antigen discovery. - Has the potential for developing vaccines against emerging/re-emerging pathogens. - Improved understanding of pathogen-host interactions, and aiding in targeted vaccine development.	- Handling and analysis of large-scale omics data needs computational resources. - Ensure the accuracy and reliability of computational predictions.	- Development of more sophisticated bioinformatics tools to handle omics data. - Continued integration of multiple omics approaches and pipelines for a holistic view of pathogen biology and translating bioinformatics findings into viable vaccine candidates respectively.
- Molecular epidemiology tools have mostly contributed to identify disease distribution, etiological agents, and outbreak tracking in tropical infectious diseases. - Recent advances have made omics tools more cost-effective and available. - Novel omics field: Lipidomics, provides insights into important environmental and genetic factors (8).	- Bioinformatic tools enhance our understanding of biological molecules (High-throughput DNA, transcriptomics, and metabolomics) and their dynamics.	- Limited optimal use of omics tools in lower income, low resource tropical environments/regions - There are some challenges in applying “One Health” approach to both disease types (communicable and non-communicable)	- Support collaborations between health experts (i.e., human and animal). - Improve capacity-building in Low and Middle Income Counties for omics research. - Concern more on ethical, privacy as well as data-sharing concerns.
- Multi-omics analysis provides comprehensive insights of biomolecules and their link to diseases. - Bioinformatics tools play a crucial role in translating omic data into meaningful conclusions (9).	Leverage ML and AI techniques for multi-omic data analysis.	- Integration of databases for multi-omics data remains a challenge.	- Further development of database integration for multi-omics analysis can be performed.
- Integration of omics data enhances pathogen detection and characterization. - ML and AI are applied to predict outbreaks of diseases and identify biomarkers. - Bioinformatics tools enable the analysis of large scale epidemiological data that aids in public health decision-making (10).	Development of novel diagnostics and targeted therapies. - Enhance personalized medicine, precision healthcare approaches, disease surveillance and outbreak predictions. - Improved understanding of pathogen evolution and resistance mechanisms.	High complexity and volume of data from different sources. - Limitations in data standardization and interoperability. - Limitations in accessing comprehensive and high-quality datasets. - Ethical and privacy concerns of utilizing sensitive health data.	Invest more in enhanced bioinformatics infrastructure and training programs. - Establish standardized data formats and essential protocols for integrating different datasets. - Support open-access data repositories and collaborative efforts in research. - Implement robust data security, privacy regulations.
Hirschsprung's disease (HSCR) is a rare developmental disorder associated with missing enteric ganglia along a portion of the intestine. - This work is applied a computational approach to identify 178 new HSCR candidate genes and two biological pathways. - Potential miRNAs were also identified as biomarkers among 137 predicted HSCR-associated miRNAs (11).	- Multi-omics network analysis provides insights into HSCR pathogenesis and biomarker identification. - Integration of various omics data can support in disease diagnosis as well as personalized medicine.	- Data heterogeneity and scalability issues show challenges in multi-omics data integration. - Molecular experiments are required for clinical validation.	- Collaborate across several disciplines for further multi-omics research. - Develop more robust computational techniques for data fusion. - Provide more priority for precision medicine applications utilizing multi-omics data.
- Multi-omics analysis involves integrating various high-throughput omics screening technologies - The review emphasizes the importance of mult-omics data integration and with some insights into ML and DL techniques for inferencing multi-omics data (5).	- Multi-omics enables detailed profiling of biological processes, supporting in disease diagnosis, prognosis, and personalized medicine. - Researchers can utilization of open-source analysis tools and databases to explore cancer, neurodegenerative diseases, aging, and drug target discovery.	- Integrating different omics data remains complex due to the issues i.e., data noise, heterogeneity, and scalability. - Validating findings and interpreting them into clinical practice require further analysis.	- In the field of aging, the major trend is the development of omics-based biomarkers, and these biomarkers can further assess multifactorial processes
- Vector-borne diseases are significant common health issues in tropical areas (mainly) affecting millions of people. There is a lack of effective drugs for these diseases for treating the patients. This work explores bioinformatics technologies to search for new, natural products having anti-vector-borne disease properties (12).	- Bioinformatics tools provide promising approaches for identifying natural products for vector-borne disease management. These technologies can analyze large volumes of datasets and predict potential drug targets.	- Complexity of identifying relevant natural products from a large biological dataset. - Validate the efficacy and safety of discovered compounds (novel) requires further research and experimentation.	- Improve collaborations to integrate bioinformatics approaches into drug discovery workflows. - Explore more on databases, molecular docking, and virtual screening to find out potential natural products. - Give more attention to conduct experimental validation of promising candidates.
- Significant progress in identifying biomarkers using omics data, mainly in cancer, etc. Key biomarkers including ANXA2, SRR, OLFML2B, and HAVCR2 were identified. These are correlated with disease prognosis and immune infiltration (13).	- Leveraging multi-omics data to enhance identifying of disease mechanisms, enhance diagnostics, and develop new targeted therapies. - ML and DL can be utilized for predictive analyses with the ntegration of various omics data to provide comprehensive insights.	- Normalizing omics data to eliminate biases while preserving biological variation is a limitation. - Issues in developing stable and interpretable biomarkers for clinical use. - Limitations in managing the complexity and volume of multi-omics data	Development of novel normalization techniques or normalization-free algorithms to enhance biomarker identification process. - Make new advancements in algorithms for multi-omics data integration. - Validation of biomarkers in clinical settings to ensure their predictive power and applicability.
- Disease specific pathways could be identified by multi-omics data integration - Finds new biomarkers and molecular mechanisms involved in tropical/other diseases. - Presents the importance of combining omics data for comprehensive analysis (14).	- Have a potential for personalized medicine as well as targeted therapies with identified biomarkers. - Improved understanding of complex diseases via integrative analysis. - Improved diagnostic accuracy as well as prognostic models.	- Requirements of high computational complexity and data management. - Integration and interpretation of heterogeneous data forms. - Requirement for robust algorithms and standardized protocols.	- Development of more efficient computational tools and algorithms for data integration process. - Improve collaborative efforts to develop large, high-quality multi-omics datasets. - Focus on translational research for applying outcomes in clinical settings.
- It is reported and validated that ALDH2 as a prognosis biomarker in early-stage of LUAD (15)	- Open the research for more investigations of biomarkers of LUAD.	- As the research on the role of ALDH2 in CSC hallmarks is limited and controversial more research is required to have a clear idea.	- Further investigations on the stem cell-related pathways of ALDH2 for LUAD treatment should be analyzed in more depth.
- Provides a review on the underlying causes of Cardiovascular Disease (CVD) and highlights possible treatment strategies by recognizing novel drug targets and biomarkers. - Reviews advances in gut microbiota studies by highlighting on how diet and microbial composition can affect the development of CVD and discusses several biological networks and other independent studies that provides support in end-stage CVD (i.e., myocardial infarction and stroke) (16)	- Opportunity is having for more investigations on the “kidney-heart” axis through a systems biology approach	- A few/no investigation in personalized treatments apart from the known heterogeneity of symptoms and risk factors for CAS and MCAO. Further approaches for the blockage of these arteries have not been studied in recent years.	- Research have to be arried out to study the dynamics of proteins from distinctly expressed genes, which can present new insights into mechanisms of atherosclerosisa - Novel research should be focused on developing tools to identify platelet variations between patients

**Table 2:** Opportunities, challenges and future recommendations of past research on utilizing suitable AI techniques for omics data processing for disease predictions (including tropical diseases).

Main findings	Opportunities	Challenges	Future recommendations
- AI improves the precision, cost-effectiveness and efficiency of gene editing, mainly in CRISPR-based technologies. - AI models can enhance the design and prediction of guide RNAs (gRNAs), that optimizes CRISPR applications. - AI provides base editing (BED), epigenome editing (epi-GED), and prime editing (PED), that improves genetic mutation corrections. - AI in precision medicine allows personalized treatments based on genetic profiles individually (17).	- AI-enabled advancements can improve the accuracy of genome editing tools significantly, and make treatments for genetic diseases effectively. - Improved AI models can streamline the gRNAs design, that increases the success rate of CRISPR interventions. - AI can support in developments of new therapeutic approaches, personalized to individual genetic makeup, thus improving patient outcomes. - Integration of AI and genome editing keeps potential for groundbreaking innovations in healthcare and biomedicine.	- Consider the ethical considerations of AI in genome editing, specifically regarding data privacy and consent. - Address the challenges in technology associated with integrating AI models with technologies in genome editing. - Overcome the high computational requirements and availability of high-quality data (for training pursose of AI models). - Manage potential effects (off-target) and ensure the safety as well as reliability of AI-enabled genome editing procedures.	- Develop standard protocols and guidelines for utilizing AI in genome editing to ensure consistency and/or safety. - Enhance collaborations between AI research community and geneticists to leverage expertise interdisciplinary. - Invest in research to enhance AI algorithms and models for accurate predictions and more efficient genome editing. - Encourage establishment of regulatory frameworks to address privacy, and ethical concerns in AI-assisted genome editing.
- PandaOmics efficiently idenstifies new and repurposed therapeutic targets and biomarkers. - This platform combines bioinformatics with multimodal deep learning for the target identification. - The model presented the ability to prioritize targets considering target-disease relationships, druggability, etc. (18)	- Usage of AI to shorten the target identification process significantly from years to months. - Facilitates the identification of high-confidence therapeutic targets for different diseases including ALS.	- Improves the identification of biomarkers for precision medicine and personalized development of therapies. - Combination and analysis of different and large-scale multi-omics data shows some specific computational challenges. - Ensuring the accuracy as well as reliability of AI-driven predictions in real-world problems. - High computational costs with the requirements for advanced infrastructure.	- Require more validation of AI-assisted targets in various disease models and clinical environments. - Requirement of constant improvement of AI-assisted models to enhance target prediction accuracy and efficiency. - Expand platform capabilities to cover more diseases and integrate various omics data types.
- Discussed the applications and advancement of Deep Learning-enabled ChatGPT/ChatBots in medical science (19)	ChatGPT's competence has a room for altering statistical process control (SPC) practices, research, and instruction.	Increased memory requirement, availability of noise in the datasets	Design and implement data standardization, establishing policies, regulations and norms, trained and educated AI-related workforce, ethical concern for data securities, and implementation challenges
- DL models have demonstrated promising success in tropical and other disease detection. - Explainable AI (XAI) methods provide more human understandable explanations on findings. - Gradient-weighted Class Activation Mapping (Grad-CAM++) is an effective class discriminative, localization method for XAI. - The study achieved high accuracy and reduced time complexity in disease detection (20).	- Improving the transparency and interpretability of AI-assisted models in medical diagnostics. - Improving diagnostic accuracy and efficiency through AI-assisted models. - Providing the predictions and identifications of critical factors in disease classification and progression. - Potential to revolutionize personalized medicine and targeted treatments	- Deep learning models’ black-box nature limits their acceptance as well as trust in clinical usage. - Issues due to the complexity of developing interpretable models without reducing the performance. - Ensure scalability and generalizability of XAI methods across various diseases is difficult. - Addressing the need for large, various, and high-quality datasets for training AI models.	- Develop more robust and interpretable AI models for good clinical integration. - Focus more on hybrid models which can balance accuracy and interpretability. - Invest more in interdisciplinary research combining AI, medical expertise, and XAI methodologies. - Encourage the sharing of complete medical datasets for AI research.
- AI techniques, particularly ML and DL, have transformed pharmacological research by enhancing efficiency, productivity, and cost-effectiveness. - AI aids in overcoming challenges in drug development with unmatched momentum (21).	- Combination of AI techniques with traditional experimental methods. - Utilization of data augmentation and XAI.	- Limitations of the availability of high-quality data. - ddressing ethical concerns is a critical requirement.	- Leverage AI's potential in pharmaceutical research.
- DL models excel in disease detection but lack of interpretability. - XAI methods such as Grad-CAM++ provide human-understandable insights. - XAI can find biases and errors in DL models. - Transparent models aid in the process of regulatory approval (20).	- Improved diagnostic accuracy and efficiency using explainable models. - Provide collaboration between AI-assisted systems and clinicians. - Has the potential to revolutionize personalized medicine and targeted treatments. - Faster and more reliable approval of AI-based medical devices.	- The black-box nature of deep learning limits clinical trust and adoption. - High complexity in creating interpretable models without performance loss. - Ensuring the scalability and generalizability of XAI methods across different diseases. - Data privacy and security concerns with handling sensitive medical data.	- Develop robust explainable AI (XAI) methods that balance accuracy and interpretability. - Invest in research for hybrid models combining AI and medical expertise. - Encourage the development and sharing of comprehensive medical datasets for AI research. - Implement stringent data privacy regulations and ethical guidelines.
- The review investigates recent developments of AI in public health and the potential challenges (22).	- Can support public health delivery through spatial modeling, controlling misinformation, surveillance in public health, risk prediction, forecasting diseases, and epidemic modeling	- To apply AI universally in healthcare is hard due to limitations in the infrastructure, lack of technical knowledge, data paucity, and privacy concerns	- Enhancing the precision and efficiency of algorithms, find solutions to moral and legal considerations, and make good standards in EHR platforms.
- AI techniques are widely applied in healthcare, aiding clinical decision support and medical imaging analysis. However, AI can be perceived as a “black box”, limiting trust. XAI aims to make AI decisions understandable by humans. - XAI can enhance understanding and trust in AI systems, crucial in life-or-death situations (23).	- XAI can improve healthcare outcomes by increasing transparency and interpretability. - It enables clinicians to comprehend AI decisions, leading to better-informed choices.	- Ensuring XAI methods are effective without compromising AI performance. - Balancing interpretability with maintaining high accuracy in complex models.	- Research avenues include developing robust XAI techniques and assessing their impact on healthcare. - Exploring alternatives to increase trust in AI beyond XAI.
- AI aids public health through spatial modeling, misinformation control, risk prediction, etc. - AI played a critical role in forecasting, contact tracing, and health care during the COVID-19 pandemic (24).	- Increased precision, effectiveness, and scalability of public health treatments.	- Limited infrastructure, technical knowledge, data paucity, and ethical problems.	- Support more developments for AI use in public health. - Encourage ethical and responsible AI practices.
- Recent advancements in AI and ML hold significant results in model-informed drug discovery and development (MID3). The IQ Consortium aims to promote the acceptance of these advanced algorithms (25).	- AI/ML-enabled analytics can improve pharmacometrics and workflows of Quantitative Systems Pharmacology (QSP). - XAI has applications in disease progression modeling, NLP aids quantitative pharmacology modeling and AI/ML provides insights into drug discovery.	- Gain knowledge of context-aware use, model explainability, and generalizability. - Problems in real-world complexity, and human-in-the-loop considerations.	Accept AI's transformative aspects while adhering to good practices for efficiency, innovation, and patient benefits.
- Though AI can be applied to various healthcare applications, its usage is limited due to being seen as a black box and XAI aims to provide more confidence by explaining model predictions (26).	- Improved trust in AI-assisted systems for healthcare applications.	- Issues in abnormalities in 1D biosignals detection. - Identifying key text in clinical notes.	- Facilitate to develop a holistic cloud system for smart cities.
AI models trained on omics data have improved the prediction performance compared to models that solely used clinical parameters. (For e.g., integrating metabolomic data significantly enhanced model accuracy for distinguishing fibrotic stages.) (27).	- Develop efficiencies in clinical trial activities (e.g., sample size reduction, improving enrollment). - Have the potential for personalized medicine in clinical settings.	- Ethical considerations related to data availability, and regulatory guidance.	- Develop novel regulatory standards and guidance for AI tools in drug development.
- XAI provides significant insights into omics data, enhancing understanding and discovery of biological mechanisms, identify novel biomarkers and therapeutic targets by identifying the relationships between omics features and diseases. This work presents the potential of XAI to interpret DL models in a better way, gaining reliability from the medical community as well as regulatory bodies. - XAI support bridging the gap between complex DL models and their real applications for identifying and treating diseases through omics data analysis (28).	- XAI improves the interpretability and transparency of DL models in omics data analysis, providing better decision-making framework in biomedical research. - Have the potential to accelerate the discovery of new biomarkers and drug targets, ultimately leads to enhanced diagnostics and personalized medicine. - Improved collaboration between computer scientists and biologists, utilizing XAI to reveal new insights from omics datasets.	- Integrating XAI with complex omics data remains a challenge (due to the high dimensionality and heterogeneity of the data) - Hard to ensure the robustness and reliability of XAI methods in different and complex biological contexts - The requirements of computational complexity and resources in DL and XAI methods can be prohibitive/lacking for some institutions. - Integrating multiple omics datasets in an interpretable way is still a challenge.	- More developments of XAI methods to satisfy specific needs of omics data analysis, ensuring user-friendlyness and accessible to the broader research community. - Improve collaboration between AI researchers and domain experts to validate XAI methods in real-world biomedical applications. - Invest in computational infrastructure as well as training programs to improve researchers’ skills needed to utilize XAI methods effectively.
- AI has been emerged as effective tool in clinical medicine, which can revolutionize different aspects of medical research including patient care (29).	- AI and clinical medicine integration has not only improved diagnostic accuracy and results of treatments, but also assisted in more efficient healthcare delivery, reduced costs, and facilitated good patient experiences.	- Limitations of AI chatbots in clinical medicine include hard management with complex histories, missing out on important information (most of the time).	- AI has shown high possibility in shaping the future of clinical medicine
- AI improves cancer diagnosis and treatment by utilizing complex omics datasets with high accuracy. - ML models identify new biomarkers and therapeutic targets, and that leads to accelerate cancer research. - AI supports in analyzing the underlying mechanisms of cancer progression and resistance. This leads to better and effective treatments (30).	- AI can be used in personalizing cancer treatment plans, (improving patient treatments and reducing side effects). - Early detection of cancer through AI- and omics analysis can drastically improve survival rates. - AI can be utilized for identifying suitable candidates and predicting responses to therapies in clinical trials.	- Issues in integrating AI into clinical workflows with high data privacy, ethical concerns, and regulatory compliance. - Issues in high computational requirements and substantial investment in technology and training for healthcare staff.	- Develop robust AI models validated across different populations and settings (for generalizability and reliability) - Enhance collaboration between AI developers, clinicians, and researchers to continuously update AI models with new data and insights. - Create XAI systems to make sure transparency and reliability among healthcare providers and patients.
- The work investigates the applications of AI, especially ML models, for risk assessment of coronary artery disease (CAD). The findings reveal the effectiveness of these models in predicting a patient's susceptibility to CAD (31).	- Earlier identification of patients at high risk of CAD supports for preventive measures. - Implementation of personalized medicine approaches for CAD prevention and treatment has enhanced the allocation of resources by focusing on high-risk patients.	- Accessing high quality and comprehensive patient data is critical for training more accurate AI models. - Interpreting how AI models arrive at their predictions is important for reliability in this technology. - Integration of AI-assisted risk assessment tools into clinical environments is necessary for widespread usage.	- Develop further research on enhancing the accuracy and generalizability of AI models for CAD risk assessment. - Implementation of automated reasoning behind AI-assisted predictions for better clinical decision-making. - Establishing guidelines for responsible use of AI in clinical risk assessment.
- AI models can learn from large datasets and identify novel insights clinicians haven't considered previously. - Augmented intelligence tools (i.e., clinical decision support systems) are important for practicing medicine safely (32).	- Integration of big data, cloud computing, and precision medicine can improve the usage of AI. - Next-generation sequencing helps personalized medicine.	- Patient data security, authentication, and validation are some challenges in AI utilization for pediatric medicine	- Pediatricians should be knowledgeable to use AI integration by understanding its potential - AI can play a crucial role in analyzing data with medical data complexity
- A summary has been provided for the repositories available on deep-learning-based lung cancer mining, and provided recent developments on deep learning models in lung cancer genomics (33)	- Comprehensive analysis of multi-omics data provides opportunities to figure-out relationships between different levels of cellular organization and provides insights into the heterogeneity in lung tumors	- The implementations of DL models for drug sensitivity and therapeutics are still having challenges and keep the potential to develop some models for predicting drug response and treatment outcome. - Multi-omics data integration from various sources require multi-step approaches and explainability.	- Personalized therapies for lung cancer patients should be explored.

#### 3.1.1 Opportunities and challenges in utilizing bioinformatics and AI techniques for tropical disease predictions with omics data

Though various Bioinformatics tools have been utilized for disease predictions with various types of datasets, there are a few research carried out for the predictions of tropical diseases using omics data types (8, 12). Apart from that, several AI based techniques have been utilized for tropical disease identification based on omics or other data types (35–37). A need analysis was conducted with 40 physicians in the work presented by (35), The predictors they have identified as the best parameters are Sodium, albumin, total bilirubin, platelets, and lymphocytes (laboratory parameters). Further, arthralgia, abdominal pain, myalgia and urine were identified as the best clinical predictors. And the common tropical infections identified in their settings were Dengue, Malaria, Leptospirosis and Scrub Typhus. Binary classification machine learning algorithms were observed providing maximum average of 79%–84% predictability in this analysis out of all ML methods used (i.e., multinomial logistic regression, multi classification ML models etc.). A review has been conducted in (36) based on 159 studies on the vector-borne diseases caused by aedes mosquito, the culex mosquito, the anopheles' mosquito, the triatome bug, the ticks, the lice, the fleas, and the blackflies etc. According to that they have recommended DL models in regular diagnostic predictions. ML and ensemble methods were reviewed in the article (37) disregarding the types of dataset utilized.

We have further reviewed the usage of advanced AI technique for the same purpose and they are presented below.

##### 3.1.1.1 Opportunities and challenges in utilizing advanced AI techniques for tropical disease predictions

Here we present opportunities and challenges in utilizing Advanced AI techniques, except the DL (CNN, RNN and Transformers) and discuss about the models: Generative Adversarial Networks (GANs), Reinforcement Learning (RL), AutoML (Automated Machine Learning), Transfer Learning, Federated Learning, and Attention Mechanisms, etc.

Although some research have focused on infectious disease forecasting with statistical learning and interpretable modeling (38) and some reviews were conducted for analyzing clinical and genomics diagnostics with AI without focusing on diseases (39), here we thoroughly review state-of-the-arts advanced AI techniques available to predict diseases (specifically tropical) and their future perspectives. Here we have classified the advanced AI technique utilized by the research into several categories: Generative Adversarial Networks (GANs), Automated Machine Learning, Reinforcement Learning (RL), Deep Transfer Learning, Federated Learning/Federated Machine Learning and Attention Mechanisms for using omics datasets for tropical and other disease predictions and presented in Table 3.

**Table 3:** Opportunities, challenges and future recommendations on utilizing other advanced AI techniques for omics data processing for tropical and other disease predictions.

Main findings	Advanced AI models utilized	Data types used	Future recommendations
- Introduced omicsGAN, which integrates two omics data and their interaction network based on generative adversarial networks - Integration of the bipartite interaction network with the multiple omics datasets can improve the outcome of disease phenotype predictions (39)	- GANs	- Multi-omics data a. The Cancer Genome Atlas Network (40), The Cancer Genome Atlas Research Network (41) b. RNA-seq mRNA expression and miRNA expression of each cancer type UCSC Xena Hub (42) c. Clinical information for each cancer type from cBioPortal (43) d. miRNA-mRNA interaction network from TargetScanHuman (44)	- Further enhancements can be conducted with the functional incorporation of the inter omics interaction network into the study
- Provides a comprehensive review on emerging innovations of GANs and gene expression data ranging synthetic gene expression data to single-cell RNA-seq analysis (from 2019 to 2023) (45)	- GANs	- Gene expression data	- Highlights the implementations of more robust and versatile GAN models to enhance the handling of multiple data types improving the low quality data
- GAN-based transcriptomics data augmentation techniques were analyzed with respect to performance indicators and classification of various cancer phenotypes (40)	- GANs	- RNA sequences - Pan Cancer Genome Atlas (TCGA) was used as the benchmark dataset	- Further validation with novel datasets may also show that various augmentation performance indicators of GANs are needed to assess its quality correctly
- Explored various Reinforcement Learning (RL) algorithms to observe solutions for the prevention of diseases and (specially Malaria) Deep Deterministic Policy Gradient (DDPG) have outperformed conventional algorithms such as Q-Learning/SARSA (41).	- RL	- No specific dataset (solutions can be utilized with omics datasets as well)	- The suitability and performance of the DDPG with omics datasets can be explored
- This work presents an AutomatedML (can be used by non-experts), which led to multiple biosignatures of better predictive performance, including reduced features and large selection of alternative predictors (42).	- Automated ML	- Proteomic, metabolomic and transcriptomic measurements of Covid-19 patients (Gene expression profiles from host/viral metagenomic sequencing of upper airway samples of Covid-19 patients, Nasopharyngeal swab samples with RNA-sequencing from Covid-19 vs. non-Covid-19 patients)	- Future work of Covid-19 analyses could use recurrent neural networks to incorporate timing information to the predictions
- This reviews limitations of AutoML for the genetic analysis of complex traits and provides an overview of several methods and some sample applications of omics datasets (43).	- Automated ML	- Omics (specially genetics data)	- Future studies can be used to implement and evaluate new AutoML methods and tools in the genetics
- Explored the capability of enhancing the prediction results for functional non-coding variants using a deep transfer learning model - It outperforms 18 state-of-the-arts computational approaches in both MPRA and GWAS (Genome-wide association studies) datasets (44).	- Deep Transfer Learning	- Genome data sets (Three MPRA and GWAS datasets)	- Explore how the cross-validation can contribute to make optimized Transfer Learning models
- Provides a review on how biological data can be used with Several DL mechanisms including Transfer learning (46).	- Deep Learning models including Transfer Learning	- Omics data types	- Though this has not been applied for specific tropical disease predictions, the prediction performances and accuracies can be explored with available datasets in the future.
- This study thoroughly evaluate the recent trends in multi-omics data analysis using DL. It highlights the current challenges in the field and discusses how enhancements in DL methods and their optimization can be used to resolve them (47).	- DL and Federated Learning	- Multi-omics data (i.e., proteins, genes and transcriptomics data)	- Recommends the implementations of new DL methodologies for data integration, which can support in disease identification and treatment. - Solutions to the problems such as overfitting, inequality, privacy protection, and lack of validation reliability, lack of interpretability raised by using DL and Advanced AI technologies such as FL Scan be further explored
- The article explains the use of FL for the diagnosis of various diseases such as cardiovascular disease, diabetes, and cancer (48).	- Federated Machine Learning	- No specific dataset, discussed mostly on clinical and image data (solutions can be utilized with omics datasets with privacy preservation and data security)	- Managing heterogeneity, privacy preservation enhancement and communication optimization can be explored more with various applications
- This work introduced a model called AttOmics, a new DL architecture including self-attention mechanism. It captures different interactions specific to a patient and the model can predict the phenotype of a patient with a limited set of parameters than a DL model (49)	- Attention Mechanisms	- The Cancer Genome Atlas (TCGA) data from Genomics Data Common (GDC) Data Portal - [AttOmics mainly works on methylation data (DNAm) and gene expression data (mRNA) but not on miRNA expression data]	- Visualizing the attention maps can further be utilized to provide new insights for a particular phenotype including tropical diseases - Investigating how to improve the performance of attention mechanisms for miRNA type of data

According to the Table 1. Following is the summary of the opportunities of utilizing Bioinformatics tools for omics data processing;

The integration of enhanced bioinformatics tools and AI-enabled platforms is improving diagnostic tools, early detection techniques, real-time monitoring, and surveillance of infectious disease pandemics. By utiliazing patient-specific omics datasets, personalized medicine techniques are being developed, which improves vaccine development by the identification of novel antigens and immune responses.

From all the existing opportunities, key points include:

• **AI for multi-omics**: Multi-omics data, combined with ML and AI techniques, can provide detailed profiling of various biological processes, facilitating disease diagnosis, prognosis, and personalized medicine.
• **Enhanced Diagnostics and Disease Surveillance**: Early detection and prediction (i.e., outbreaks) can be improved through advanced diagnostic tools and surveillance methods.
• **Personalized Medicine**: Genetic profiles can be utilized in tailoring personalized treatments, with multiple omics data by enabling personalized pharmaceutical approaches and optimizing therapeutic strategies.
• **Vaccine Development**: Comprehensive antigen discovery has advanced vaccine design, including both emerging and re-emerging pathogens, improving our knowledge of pathogen-host interactions.
• **Novel Diagnostics and Therapeutics**: The identification of new biomarkers and drug targets supports the development of novel diagnostics and targeted therapies.
• **Understanding Pathogen Evolution**: Insights on pathogen evolution and resistance mechanisms can be enhanced.
• **Open-Source Tools and Databases**: Researchers can use the available resources to explore diseases such as cancer, neurodegenerative diseases, and aging, and to discover drug targets.
• **Natural Product Identification**: Bioinformatics tools are good for the identification of natural products for handling vector-borne diseases.
• **Improved Diagnostic Accuracy**: Integrative analysis of different omics datasets leads to more diagnostic accuracy and prognostic models.

To summarize, the integration of multiple omics data and more advanced bioinformatics is providing significant advancements in disease diagnosis, personalized medicine, vaccine development, and identifying and gain knowledege of complex diseases.

According to the Table 1. following is the summary of the limitations and challenges of utilizing Bioinformatics tools for analyzing and integration of omics datasets

• **Data Complexity and Variability**: High complexity and variability of omics dataset require advanced computational methods for comprehensive analysis. The integration of different datasets has limitations due to issues such as data noise, scalability, and heterogeneity.
• **Ethical and Privacy Concerns**: Handling sensitive health datasets involves important ethical considerations, including privacy issues.
• **Access and Infrastructure**: There exist limitations in accessing annotated, high-quality, data for training AI-assisted models. Several regions, including lower-income and low-resource tropical areas, facing constraints in infrastructure and technical support.
• **Cost**: The high expenses of sequencing and data analysis leads limiting widespread application.
• **Data Standardization and Interoperability**: Issues in data standardization and interoperability slow down the integration process of diverse datasets.
• **Computational Resources**: Limitations in processing and analyzing huge omics datasets i.e., requirements of enough computational resources and robust algorithms.
• **Experimental Validation**: Some molecular experiments are required for clinical validation of findings, and converting these findings into clinical practice needs more analysis.
• **Clinical Application**: Issues in developing stable and interpretable biomarkers for clinical purpose is complex, and validating the efficiency and safety of newly discovered biomarkers requires more research and experiments.
• **“One Health” Approach**: Utilizing the “One Health” approach to communicable and non-communicable diseases shows some challenges.
• **Natural Product Identification**: Identifying relevant natural products from huge biological datasets and validating their efficacy, reliability and safety needs further research.
• **Proper Data Management**: Managing the complexity of high volume multi-omics data, ensuring their accuracy and reliability of computational predictions, and normalizing omics data to remove biases while protecting biological variation are some limitations.

To summarize, the successful integration and application of omics data require some improved computational methods, good analysis, ethical considerations of data, sufficient infrastructure, and enough research efforts for validation and clinical developments. Further, if the real-world applicability of these opportunities considered, mainly the heterogeneity and the complexity of the omics data play a major role. While doing the integration, this complexity can lead in several difficulties to integrate datasets in a meaningful way. For example if we try to integrate proteomics and transcriptomics data for an analysis of a specific disease, due to the complexity of the data it prevents from meaningful analysis. It may need some advanced computational techniques and careful normalization techniques to overcome this issue. Without proper handling of this, it can result in incomplete and biased analyses. Furthermore, there is a significant challenge in real world usage of omics data due to different data formats available or lacking of a standardized data formats for data storage. Firstly, this can be resolved by adopting researchers to utilize universal data formats of omics data. Then, more expert knowledge and knowledge dissemination can solve this problem to some extent by supporting the implementations of metadata standards. Further, development of data conversion tools and pipelines is also required in some instances. For instance, some bioinformatics platforms Galaxy, Bioconductor, and Nextflow provide integrated environments. They can process multiple formats and transform into compatible formats through workflows. As most of the AI models act as black boxes, lacking of interpretability is a major issue in understanding the rationale behind the clinical settings. Therefore, it should be managed with the collaborations between the experts in the fields. Further, explainable AI can be utilized with omics data sources to resolve this problem. LIME (Local Interpretable Model-agnostic Explanations) and SHAP (SHapley Additive exPlanations) are two famous model-agnostic methods that can be used to interpret predictions made by the current complex AI models such as Deep Neural Networks or transformers. In addition to this, as the omics data is highly sensitive they may limit the availability of the data for research purposes or ethical clearance should be received in order to utilize the data for AI model training and validation. To address the challenges of infrastructure and omics data accessibility in tropical and resource-poor regions, funding support can be provided by encouraging the collaborations between governments as well as deploying the low cost technology and infrastructure in resource- constrained settings. Further, leveraging cloud computing for omics data access can be facilitated for these regions to overcome this issue. Furthermore, organizing training programmes and workshops for the local universities and other educational institutions may enhance the technical expertise in the region as well.

According to the Table 2. AI-enabled advancements in omics data for tropical disease identifications are significantly improving different aspects of genome editing, personalized medicine, and healthcare. Major points include:

• **Genome Editing and CRISPR**: AI-assistnce enhances the accuracy of genome editing tools and speed up the design of guide RNAs (gRNAs). This increases the success rate of CRISPR interventions and makes treatments for genetic diseases more effective and success.
• **Therapeutic Development**: AI assists the design and implementation of new therapeutic approaches personalized to individual genetic makeups. This improves patient outcomes.
• **Innovation in Healthcare**: The integration of AI-assisted genome editing holds the possibility for groundbreaking innovations in healthcare/biomedicine, accelerating target identification and analysis processes from years to months. It facilitates the identification of high confidence therapeutic targets for several diseases, including ALS.
• **Precision Medicine**: AI enhances the biomarker identification for precision medicine and the personalized therapy development. It improves the diagnostic accuracy and efficiency via XAI models.
• **Pharmacometrics and Quantitative Systems Pharmacology (QSP)**: AI and ML based identifications enhance pharmacometrics and workflows of QSP. It further provides insights into drug discovery and improves reliability of AI-assisted systems for healthcare tools and applications.
• **Personalized Medicine**: AI-assisted models accelerate the identification of novel biomarkers and drug targets. It further leads to improve the diagnostics and personalized medicine. Furthermore it helps in personalizing cancer treatment plans by improving patient treatments, and reducing their side effects.
• **Medical Diagnostics**: AI-assisted models enhance diagnostic accuracy as well as efficiency, providing critical analysis on predictions and identifications for disease classification and progression, and bringing up collaboration between AI systems and clinicians.
• **Public Health**: AI-assisted models support public health delivery through spatial modeling, disease forecasting, misinformation control, risk prediction, surveillance, and epidemic modeling. XAI further enhances healthcare effects by providing high level of transparency and interpretability, allowing clinicians to make better choices.
• **Clinical Trials**: AI-assisted models can speed up clinical trial tasks, such as sample size reduction and improvement of enrollment, and can identify matching candidates and predict responses to different therapies.
• **Big Data and Cloud Computing**: The integration of big data, cloud computing, as well as precision medicine improves the usage of AI, with the support of next-generation sequencing for personalized medicine.

In summary, AI-assisted models can revolutionize personalized medicine and targeted treatments, improving accuracy and efficiency of diagnostics and healthcare delivery, and bringing up collaboration between computer scientists and biologists to reveal novel insights from various omics datasets.

Validating AI-assisted models such as transformers and DL based models that use omics data in medical and clinical settings requires precise evaluation methods and techniques to ensure they are effective and secure for use. Though these AI models have the opportunities to revolutionize healthcare, validation in real-world clinical or field settings is crucial and a must to receive regulatory approval. Validating AI-assisted models using omics data, in clinical or field settings needs extensive testing across diverse datasets and environments. It requires both retrospective and prospective validation to verify that models are accurate, generalizable, and capable of improving patient outcomes. To this end, regulatory approval, ethical compliance, and real-world testing are some of the major entities of the validation process. As AI emerges with the time, the implementation of robust validation frameworks is important integration of these technologies into healthcare successfully.

According to the Table 2. Limitations and challenges of AI-enabled advancements in omics data for tropical disease identifications are:

**Ethical Considerations:** Ensure data privacy and receiving proper ethical consent are critical.
**Technological Integration:** Integrating AI-assisted models with genome editing technologies shows some significant challenges.
**Computational Requirements**: High computational nrequirements and the need of high-quality data for AI model training are essential.
**Safety and Reliability:** Controlling off-target problems and ensuring the safety and reliability of AI-assisted genome editing procedures are very much needed.
**Multi-Omics Data:** Integrating and analyzing large-scale multi-omics data provides some computational limitations and challenges.
**Model Accuracy:** Accuracy and reliability of AI-assisted predictions in real-world scenarios is critical.
**High Costs and Infrastructure**: High computational costs, infrastructure requirements, and data quality issues are important and should be addressed.
**Model Interpretability**: DL models limit their clinical acceptance and trust. Developing interpretable models without performance loss is complex.
**Scalability and Generalizability**: Problems exist in ensuring scalability and generalizability of explainable AI (XAI) models across different diseases.
**Clinical Integration**: Combining AI into clinical workflows involves in high data privacy, regulatory compliance, ethical considerations, and considerable investment in technology advancements and training for healthcare professionals.
**Data Quality and Security**: Accessing high-quality datasets, ensuring patient data security and managing computational complexity are critical.
**AI in Clinical Practice**: AI chatbots face difficulties to handle complex patient histories and ensuring more comprehensive information gathering.

In summary, addressing these challenges is important to succeed in integration of AI in genome editing as well as in healthcare. According to the Table 4, we have identified significant opportunities in Vision Transformer (ViT) based Implementations for Omics Data Processing for disease predictions. It includes;

**Table 4:** Opportunities, challenges and future recommendations of past research on utilizing vision transformer (ViT) based implementations for omics data processing for disease predictions.

Main findings	Opportunities	Challenges	Future recommendations
- The rapid advancement of DL, specifically transformer-based architectures and attention mechanisms, have had considerable implications in genome data analysis. These techniques, initially successfully used in NLP, have been applied to genomic data (50).	- Utilizing transformer architectures and attention mechanisms to improve genome and transcriptome data analysis.	- Critically evaluate the advantages and limitations of DL techniques in the context of genome data analysis.	- Assessing on the future direction of research in DL methodologies for genomics continuously.
- Transformer-based models (i.e., BERT and GPT-3), have been effectively applied to handle bioinformatics data, including sequence analysis, structure identification, and functional genomics (51).	- Transformer based models offer possible improvements in predictive accuracy, interpretability, and generalizability over various bioinformatics applications. Further, they provide a unified approach for integrating multi-omics data.	- Requirements of large annotated datasets, high computational resources for training, and the problems in adapting these models to bioinformatics problems are challenges.	- Future research focus on developing domain-specific models, improving model efficiency, and developing comprehensive datasets. - Collaboration between biologists and AI researchers is crucial for improving the field.
- The GeneViT model uses Vision Transformers (ViTs) and Improved DeepInsight methods for cancer classification using gene expression data effectively (52).	- The integration of ViTs with gene expression data can lead to more accurate cancer diagnoses. This offers potential for developing personalized treatments based on cancer classification.	- High computational requirements and the requirements for annotated datasets are challenges. - Developing models suitable for clinical settings requires thorough validation and regulatory approvals.	- Optimizing computational efficiency, extend the availability of annotated datasets, and performing more clinical trials to validate the model's efficacy in real settings.
- Transformer-based models can effectively integrate multiple omics data with cancer pathway details, enhancing the accuracy of cancer predictions (53).	- Enhances personalized cancer treatment by providing precise classifications and predictions. - It further allows for the integration of different types of omics data and providing a comprehensive view of cancers.	- Increased computational costs, the necessity for extensive annotated datasets, and the complexity of integrating multiple omic data types. - Interpreting the model and maintaining the clinical relevance is also challenging.	- Support in improving computational efficiency, creating bigger and more diverse annotated datasets, and ensuring strong validation of models in clinical settings. - Collaboration between computational and clinical researchers is essential.
- AI and ML can significantly improve predictive medicine through applications like early disease detection, personalized treatments, and patient results predictions (54).	- AI can improve diagnostic accuracy, streamline clinical workflows, and facilitate personalized medicine by combining various, large datasets.	- Problems in data privacy concerns, high costs for computational resources, requirement of large annotated datasets, and the complexity of integrating AI systems into clinical practice.	- Mainly focusing on interdisciplinary collaboration, strong validation of AI models in clinical environments, and continuous development of ethical guidelines for AI-based healthcare applications.
- Transformer-based models utilized in results in various bioinformatics processes show significant results (51).	- These models can enhance multiple omics data integration, improving disease prediction as well as diagnosis, and accelerating drug discovery via accurate and thorough data analysis.	- The requirement for large and high-quality datasets, high computational costs, and the interpretation difficulties of complex model predictions.	- Focus more on developing more efficient models, improving interpretability, and making standardized benchmarks for bioinformatics applications.
- Vision Transformers (ViTs) offer outstanding improvements in medical image analysis tasks outperforming traditional CNNs (55).	- ViTs can utilize large datasets to enhance diagnostic accuracy, provide early disease detection, and integrate multi-modal medical data for more detailed analysis.	- High computational requirements, requirement of large and annotated datasets, difficulty in model interpretability	- Focus more on enhancing computational efficiency, improving model interpretability, and validating ViTs in different clinical environments.

Transformer architectures and attention mechanisms have various significant opportunities in bioinformatics data analysis including genomics and transcriptomics. These models can improve interpretability, accuracy, and generalizability. Further, they offer a unified approach for integrating multi-omics data. By integrating recent Vision Transformers (ViTs) with gene expression data, cancer diagnoses can be conducted with significant accuracy, enabling personalized cancer treatments via precise classifications and predictions. In addition, transformers allow comprehensive cancer analysis with the integration o various types of omics datasets. AI further enhances diagnostic accuracy, provides clinical workflows, and facilitates personalized medicine by utilizing large volumes of datasets. These models can improve the process of multi-omics data integration, disease prediction, and diagnosis, and further accelerate drug discovery via data analysis. ViTs are utilized in improving diagnostic accuracy, enabling early disease detection, and integrate multi-modal medical data for detailed analysis.

DL techniques for genome data analysis provide several advantages with notable limitations. These techniques can improve predictive accuracy and reveal complex patterns in large datasets. However, they request large and annotated dataset and high computational resources for training, which can be challenging to obtain. Integrating DL models into bioinformatics applications and clinical environments necessitates thorough validation and regulatory approvals. The complexity of integrating multiple omic data types and high computational costs are significant bottlenecks. Interpretation of the model outputs and keeping track of clinical relevance also haven significant limitations. In addition, data privacy concerns, high costs for computational resources, and the difficulty of integrating AI systems into clinical practice further complicates the utilization of DL models.

According to the Table 3, we observed various space of improving the existing models and following observations and highlights were made for future research improvements. This is one of our main contributions in this review as well.

- Implementation of more robust and versatile GAN meodels may allow handling multi-omics data/multiple data types improving the low quality data
- Assessing the quality of GANs in a correct way requires various augmentation performance indicators
- The applicability and the performance of Deep Deterministic Policy Gradient (an advanced Reinforcement algorithm) with omics data have to be explored
- Managing heterogeneity, privacy preservation improvements and communication optimization can be explored more with various datasets and applications using Federated Machine Learning
- Exploration of cross-validation contribution to make optimized Transfer Learning models can be observed
- Attention mechanisms can be utilized with attention maps to visualize more on how omics data can be used to do accurate predictions of tropical diseases

## 4 Results and conclusion

Based on our comprehensive review (under Table 1) we observed the following main findings (summary) of utilizing Bioinformatics tools for Omics Data Processing for tropical disease predictions.

1. The integration of multiple omics data improves biomarker identification, disease predictions including disease outbreak predictions, such as in the study of Hirschsprung's disease (HSCR) and vector-borne diseases. Further, it provides treatment methods from the support of ML and AI techniques.
2. Bioinformatics resources like GenBank, Uniprot, EuPathDB, and tools such as BLAST and ClustalW, Clustal Omega are commonly used in infectious disease research. They assist a lot in significant advancements.
3. Bioinformatics tools and techniques lead to considerable progress in pathogen characterization, vaccine development, monitoring progression of pathogen evolution, and host responses in infectious disease research.
4. Integration of multi-omics data assists scientific community to identify new relationships between biomolecules and disease-phenotypes. Further, it may establish biomarkers in detail, as well as investigate signaling pathways.
5. The integration of bioinformatics with genomics and proteomics data speed up the processes of antigen discovery, utilizing high-throughput techniques to identify potential vaccine candidates.
6. Molecular epidemiology tools can mainly contribute to identify disease distribution, etiological agents, and outbreak tracking, importantly in tropical-infectious diseases.
7. Recent advances in omics technologies have made them more accessible and cost-effective, with new fields like lipidomics offering insights into environmental and genetic factors.
8. Some Bioinformatics tools play a critical role in translating omics data into meaningful interpretations and conclusions. This enables the analysis of large-scale epidemiological data to support in public health decision-making.

According to our comprehensive review (under Table 2) we observed the main findings (summary) of utilizing AI assisted techniques for Omics Data Processing for tropical disease predictions. AI-assisted models can improve the precision, cost-effectiveness, and efficiency of CRISPR-based gene editing, optimizing gRNA design, and enabling advanced genetic correction techniques (i.e., base, epigenome, and prime editing). Several existing platforms like PandaOmics utilize AI-assisted models to identify and repurpose therapeutic targets and biomarkers efficiently. DL models have shown promising results in disease identification, with XAI methods like Grad-CAM++ providing human understandable insights by further improving model transparency. AI models has significantly impacted pharmacological research, supporting drug development, public health efforts, and personalized medicine by integrating large datasets for better predictions and diagnoses. However, we still have challenges in data privacy, high computational costs, and model interpretability, which XAI aims to address, improving trust and regulatory approval in several clinical applications.

According to our comprehensive review (under Table 4) we observed the main findings (summary) of utilizing vision transformers and computer vision assisted techniques for Omics Data Processing for tropical disease predictions. The novel and rapid advancements of deep learning, mainly transformer-based architectures and attention mechanisms, have significantly impacted omics data analysis including genome data analysis. Transformer-based models, i.e., BERT and GPT-3, have been mainly applied to bioinformatics data, including sequence analysis and functional genomics. The GeneViT model, utilizing Vision Transformers (ViTs), has shown effectiveness in cancer classification using gene expression data. These models enhance the accuracy of cancer predictions by integrating multiple omics data with cancer pathway details. Additionally, AI and ML applications in predictive medicine, such as early disease detection and personalized treatments, have shown significant improvements.

## 5 Future directions

Future perspectives for genome data analysis and precision medicine highlight the need for standardized protocols and open-access data sharing to ensure consistency and reproducibility. Investing more in computational settings, infrastructure and AI-assisted Bioinformatics training programs is crucial to build capacity and enhance Bioinformatics and AI applications. Motivating collaborations between Bioinformaticians, clinicians, and other stakeholders can bridge the gap between practical usage and omics data analysis. Ethical concerns, data privacy, and regulatory frameworks should be established to address concerns in AI-enabled genome editing. Developing more sophisticated and interpretable AI models, particularly hybrid models that can balance accuracy and interpretability, will facilitate better clinical integration. Further, open-access databases and collaborative research environments should be promoted to enhance multi-omics integration and applications in precision medicine. Recent advancements in algorithms and computational methods for data fusion and normalization are necessary to handle the complexity of omics data. Utilizing interdisciplinary research and ensuring robust validation of AI-assisted models in clinical settings can improve their reliability as well as generalizability. AI-driven approaches can be integrated into drug discovery workflows, highlighting experimental validation of significant candidates. Furthermore, enhancing AI's role in public health and utilizing ethical AI practices may support the broader adoption of AI-assisted models in healthcare.
